# Long-term Effects of Snake Envenoming

**DOI:** 10.3390/toxins11040193

**Published:** 2019-03-31

**Authors:** Subodha Waiddyanatha, Anjana Silva, Sisira Siribaddana, Geoffrey K. Isbister

**Affiliations:** 1 Faculty of Medicine and Allied Sciences, Rajarata University of Sri Lanka, Saliyapura 50008, Sri Lanka; subodhawaid@gmail.com (S.W.); nkanjanasilva@gmail.com (A.S.); sisira.siribaddana@gmail.com (S.S.); 2 South Asian Clinical Toxicology Research Collaboration, Faculty of Medicine, University of Peradeniya, Peradeniya 20400, Sri Lanka; 3 Clinical Toxicology Research Group, University of Newcastle, Callaghan, NSW 2308, Australia

**Keywords:** long-term, chronic, delayed, envenoming, snakebite

## Abstract

Long-term effects of envenoming compromise the quality of life of the survivors of snakebite. We searched MEDLINE (from 1946) and EMBASE (from 1947) until October 2018 for clinical literature on the long-term effects of snake envenoming using different combinations of search terms. We classified conditions that last or appear more than six weeks following envenoming as long term or delayed effects of envenoming. Of 257 records identified, 51 articles describe the long-term effects of snake envenoming and were reviewed. Disability due to amputations, deformities, contracture formation, and chronic ulceration, rarely with malignant change, have resulted from local necrosis due to bites mainly from African and Asian cobras, and Central and South American Pit-vipers. Progression of acute kidney injury into chronic renal failure in Russell’s viper bites has been reported in several studies from India and Sri Lanka. Neuromuscular toxicity does not appear to result in long-term effects. Endocrine anomalies such as delayed manifestation of hypopituitarism following Russell’s viper bites have been reported. Delayed psychological effects such as depressive symptoms, post-traumatic stress disorder and somatisation have been reported. Blindness due to primary and secondary effects of venom is a serious, debilitating effect. In general, the available studies have linked a clinical effect to a snakebite in retrospect, hence lacked accurate snake authentication, details of acute management and baseline data and are unable to provide a detailed picture of clinical epidemiology of the long-term effects of envenoming. In the future, it will be important to follow cohorts of snakebite patients for a longer period of time to understand the true prevalence, severity, clinical progression and risk factors of long-term effects of snake envenoming.

## 1. Introduction

Snakebite is a neglected tropical disease that mainly affects poor farming communities in the rural tropics [1]. Snake envenoming is often under-reported, and there is limited accurate data on the global burden of snakebites. The high estimates suggest that there are 5.5 million bites, 1.8 million envenomings and 94,000 deaths annually due to snakebite [2]. The largest burden of snakebite occurs in South Asia, Southeast Asia, Sub-Saharan Africa and Latin America. 

Snake envenoming can cause acute local and systemic effects due to the actions of toxic components in the venom. Some elapid and viperid snakes cause tissue injury at the bite site, manifesting initially with oedema, pain, redness and blistering. In more severe cases, there may be subsequent dermonecrosis and myonecrosis, occasionally requiring debridement and rarely amputation. The commonest important systemic manifestations of snake envenoming are venom induced consumption coagulopathy, neuromuscular paralysis, acute kidney injury, myotoxicity and cardiovascular collapse [3,4,5,6]. Initial treatment is with antivenom and supportive care, depending on the specific clinical effects. The vast majority of patients are then discharged once these effects have resolved. Occasionally patients with complications require a more prolonged hospital stay. 

Most snakebite patients are not followed up once they are discharged from hospital and the acute effects have resolved. They rarely have further contact with the healthcare system in relation to the snakebite. Although some acute pathological effects of envenoming might completely resolve within a few days of the bite, other pathological effects or their consequences may last for months or years [7,8,9,10]. However, due to the lack of follow up clinically and in research studies, the long-term effects of snake envenoming are poorly defined. In addition, some effects, such as the psychological effects resulting from the snakebite, are likely to have a delayed onset [11]. 

In this review, we aim to summarise our current knowledge of the long-term effects of snake envenoming and identify the knowledge gaps.

## 2. Long-term Sequalae of Local Effects

### 2.1. Local Necrosis Resulting in Amputation

Most viperid and some elapid envenomings cause local tissue injury. Occasionally, this is more severe with varying degrees of necrosis of the skin, subcutaneous tissues and muscles [12,13]. In addition to the toxin mediated tissue necrosis, rapidly developing oedema can lead to a compartment syndrome, which can result in limb ischaemia [14]. Secondary infections at the bite site can further aggravate the tissue injury and prolong the recovery [15]. The above conditions usually require surgical management. Uncommonly, partial amputation of the limb at different levels and/or digits is required in order to stop further spread of tissue injury [16]. Even if amputation is not required, tissue loss resulting from necrosis, subsequent fibrosis and formation of contractures of various tissues can lead to impaired or loss of function in limbs. 

Detailed descriptions of the long-term sequalae of the local effects of authenticated snake envenoming are rare. In particular, literature on the long-term socioeconomic burden following amputations due to snake envenoming is scarce. Several studies report the acute stages of snakebites that result in amputation. This provides some insight into their long-term impact. In a study from Nigeria, of 16 snakebite patients who presented late and lost an upper or lower limb due to amputation, the median age was 12 years (2–55). This demonstrates the duration of the disability in the survivors, being so young at the time of the bite [16]. Cobras [13,16,17,18,19], true vipers and pit-vipers [20,21,22,23,24,25] are reported most commonly to cause extensive local tissue injuries. In a recent population-based cross-sectional study from Sri Lanka, of 816 snakebite victims, 26 (3.2%) had a range of musculoskeletal disabilities that persisted for an average period of 13.4 years [9]. Despite the limitations of a population-based study, in which case-authentication is lacking, the study reported a range of long-term disabilities due to local envenoming following snakebite. These included contractures and deformities, muscle wasting, joint stiffness, reduced range of movement and impaired balance. Some of these effects, such as reduced range of movements are mostly reported in those who did not undergo an exercise program to improve the range of motions, hence likely preventable [9].

### 2.2. Chronic Ulcers

Another important local injury is the development of a chronic ulcer at the bite site. This can cause extensive scarring followed by transformation into squamous cell carcinoma, similar to a Marjolin ulcer. Chronic ulcers have been reported in a possible pit-viper bite in Brazil [26] and a black-necked spitting cobra (*Naja nigricollis*) bite [27]. These consequences are extremely rare, but can result in severe morbidity.

### 2.3. Chronic Local Pain and Swelling

Less severe effects of local envenoming have also been reported for some snakes. In a follow-up telephone survey conducted in California, 6 of the 13 patients with rattlesnake bites reported localised pain, numbness or paraesthesia, abnormal skin peeling and discolouration at the bite site, with persistent weakness of the bitten extremity for 7 months to 12 years [28]. Similar effects were experienced for weeks to months after the bite by snakebite survivors in Sri Lanka [10]. Complex regional pain syndrome has been reported following viper bites from South Korea, Turkey and Norway [29,30,31]. This symptom complex lasts over six weeks and includes allodynia and hyperalgesia, in addition to pain at the bite site. The burden of persistent local pain and its impact on the post-bite quality of life requires further investigation, as it is likely that many snakebite survivors experience such, without seeking medical care. Persistent swelling in 27 of 145 patients who had Malayan pit-viper (*Callocellasma rhodostoma*) envenoming has been reported from Thailand [32].

**Figure 1:**
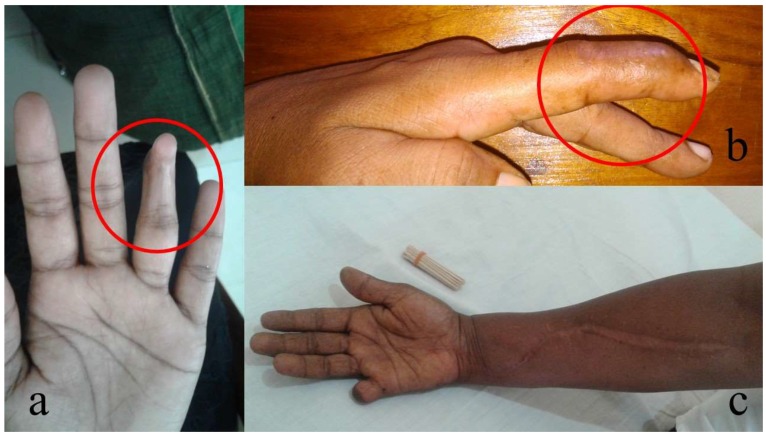
Various long-term local effects of the bites by Merrem’s hump-nosed pit viper (*Hypnale hypnale*) in Sri Lanka: (**a**) a contracture deformity involving the distal interphalangeal joint of the ring finger in left hand (red circle); (**b**) a contracture deformity involving the distal interphalangeal joint of the index finger in left hand (red circle); and (**c**) amputation of the right little finger due to local necrosis with the fasciotomy due to compartment syndrome of the right forearm. (All photographs are published with permission of the patients).

Therefore, not only the consequences of severe local effects, but also the burden due to mild long-term local effects are important in snakebite survivors and need to be addressed (Figure 1).

### 2.4. Blindness due to Primary Venom Effects

Venom ophthalmia from African and Asian spitting cobras is uncommonly reported in humans [33]. In the reported cases, painful conjunctivitis usually resolves in days. However, if left untreated, corneal ulceration and leukoma may lead to permanent blindness, especially in cases of *Naja nigricollis* [33,34].

## 3. Chronic Kidney Disease

A range of snakes have been reported to cause snakebite associated acute kidney injury, including Russell’s viper (*Daboia russelii* and *D. siamensis*) [35,36,37,38] carpet vipers (*Echis* spp.) [39], the lance-headed pit-vipers (*Bothrops* sp.) [40,41] and rattlesnakes (*Crotalus* sp.) [20,42], as well as some Australasian elapids such as brown snakes (*Pseudonaja* sp.) [43], taipans (*Oxyuranus* sp.) [44] and tiger snakes (*Notechis* sp.) [45]. The mechanism of acute kidney injury in snakabite remains unclear, but is most likely due to secondary effects including hypotension, thrombotic microangiopathy, immunological reactions, although direct nephrotoxicity is still considered a possible mechanism [3,6]. In most cases, the acute kidney injury resolves after treatment with antivenom and supportive care, with or without dialysis. Progression to chronic kidney disease has been reported in a few studies [7,46,47,48,49]. 

In an observational study of 54 patients from Sri Lanka with acute kidney injury following snake envenoming, 34 regained normal renal function after one year and 20 (37%) developed chronic kidney disease [7]. Of the patients who developed chronic kidney disease, five had end stage kidney disease, four had stage 4 and eleven had stage 3 chronic kidney disease. The duration of renal replacement therapy during the acute kidney injury and persistence of high creatinine concentrations after the acute stage had resolved, were the best predictors of chronic kidney disease. Glomerular sclerosis and interstitial lymphocytic infiltration, tubular atrophy and scarring were the commonest histopathological changes on renal biopsy. The major limitation of this study was that no patients had baseline creatinine concentrations done prior to the snakebite, hence the possibility of chronic kidney disease prior to the snakebite cannot be excluded. In addition, the biting species was only confirmed in half of the cases. The study is therefore likely to have significantly over-estimated the frequency of chronic kidney injury following snakebite. 

An observational study from India reported 42 patients with acute kidney injury, including five shown to have acute interstitial nephritis on renal biopsy. Of the 42, four developed chronic kidney disease stage 3 to 5D, 4–10 months after the bite [48]. All four of them had severe acute kidney injury following the bite with a prolonged hospital admission. In the same cohort, of eight patients who had histological findings of acute tubular necrosis during the acute stage, all recovered early and regained normal renal function. The absence of baseline creatinine, lack of snake authentication and lack of uniformity of the follow-up periods were major limitations of the study. Although the study suggested that the acute interstitial nephritis following snake envenoming was more likely to progress into chronic kidney disease, the absence of information prior to the bite means that it was unclear whether the patient was not already predisposed to or had pre-existing chronic kidney disease.

A cohort study from India of 60 patients who had snakebite associated acute kidney injury that required dialysis, found persistent renal dysfunction, proteinuria, and/or hypertension in 24 (40%) patients, after a mean follow up period of 45 months [46]. Of these, three patients progressed to end-stage renal disease. This study provides a better estimate of the frequency of chronic kidney injury, although it reports this only for those with a severe acute kidney injury at the time of the bite. In another study from India of 100 patients who developed acute kidney injury following snake envenoming, eight patients were found to have “chronic renal failure” after six months [47].

In a 10-year follow-up of a cohort of 37 children in India with acute kidney injury, there were three patients in which the acute kidney injury followed a snakebite. They had either proteinuria or hypertension, proteinuria and increased estimated glomerular filtration rate [49]. 

The above studies suggest that, in some patients with acute kidney injury following snake envenoming, there is a risk of persistent renal dysfunction over months to years, which may ultimately progress to chronic renal failure. However, there are a number of inherent limitations in the above studies, including no information on renal function prior to the snakebite (i.e., pre-existing chronic kidney disease), authentication of the snake species, lack of uniformity in the follow-up periods, lack of standardisation of the measure of the clinical severity of the initial acute kidney injury and limited details of the interventions used during the acute stage. It is therefore difficult to determine the prevalence, predictors and time scale of chronic kidney disease that is a result of snake envenoming.

## 4. Neurological Effects

### 4.1. Neuromuscular Paralysis

Neuromuscular paralysis is one of the major systemic effects of elapid snake envenoming, including bites by kraits (Genus: *Bungarus*), cobras (Genus: *Naja*), taipans (Genus: *Oxyuranus*), tiger snakes (Genus: *Notechis*) and some vipers, such as Russell’s viper (*Daboia russelii*) [5]. Snake venom neurotoxins primarily affect the neuromuscular junction and disrupt transmission across the neuromuscular junction. Clinically, this manifests as a rapidly progressing, flaccid paralysis that initially involves extraocular and facial muscles, gradually descending to bulbar, neck, respiratory and limb muscles. Of the two major groups of neurotoxins, pre-synaptic neurotoxins enter the motor nerve terminal and lead to a depletion of synaptic vesicles followed by destruction of the motor nerve terminal. Recovery is via natural repair of the motor nerve terminal, which initiates over 3–5 days but may take several more days for complete repair to occur [50,51]. Snakes such as kraits, taipans and tiger snakes have venoms rich in pre-synaptic neurotoxins. In contrast, post-synaptic or alpha neurotoxins (three-finger toxins) antagonise the nicotinic acetylcholine receptors in the motor end plate and lead to a “curare-like” neuromuscular block, which is reversible in comparison to pre-synaptic neurotoxins [52]. Many elapid snakes have alpha neurotoxins in their venom [53]. Recently, this group of toxins have been shown to be clinically less important [52]. 

Most observational studies have shown that the neuromuscular paralysis in snake envenoming completely resolves within several days [19,54,55], based on clinically observed neurological features. In a cohort of 33 patients with authenticated Indian krait (*Bungarus caeruleus*) bites in Sri Lanka, which measured serial single-fibre electromyography, patients continued to have sub-clinical neurotransmission anomalies after clinically apparent paralysis resolved [56]. These electromyographic abnormalities consisted of increased neuromuscular jitter and increased neuromuscular blocks. These were severe in the acute period and then gradually resolved. Mild neurotransmission abnormalities were still present at six weeks after the krait bite, but were absent after six months. No venom was detected in patients’ blood after the first dose of antivenom. This suggested that the neurotransmission abnormalities are irreversible after pre-synaptic neurotoxin mediated damage, and take several weeks to fully recover, rather than there being delayed venom effects.

Another study recruited 26 patients who had a neurotoxic snakebite in the previous year. The study found abnormal nerve conduction parameters in all patients, including prolongation of sensory, motor and F-wave latencies and reduction of conduction velocities, when compared to 22 control subjects [57]. None of the patients had any neuromuscular transmission abnormalities. The study concluded a possible sub-clinical demyelinating type polyneuropathy due to neurotoxic envenoming. However, this study identified the patients after the snakebite and therefore, lacked species-authentication, details of the clinical severity of the neuromuscular paralysis and venom concentrations during the acute stage. This limits any conclusion in associating the neurophysiological anomalies with the snake envenoming. 

There is a report of persistent unilateral ptosis due to paralysis of the frontalis muscle in a patient bitten by a European adder (*Vipera berus*) [58]. The patient had severe swelling over the frontalis muscle for several days, so it is unclear whether the weakness of the frontalis was due to direct muscle injury or not.

### 4.2. Neurological Effects Secondary to Hypoxic or Ischemic Events

Permanent neurological injury from hypoxic encephalopathy is an important long-term effect of snake envenoming. Respiratory paralysis or cardiac arrest can both result in hypoxia and multiorgan failure. In many cases, this results in an early death, but some patients survive with significant neurological impairment. A single case report described the persistence of cerebellar ataxia in a patient who developed severe neuromuscular paralysis following a suspected Indian krait (*Bungarus caeruleus*) bite [59]. The ataxia was clinically apparent after the patient had recovered from the severe neuromuscular paralysis, two weeks after the bite. The report did not provide sufficient details to exclude pre-existing ataxia. The patient was lost to follow up after two months and the possible pathophysiological mechanisms are difficult to determine. Ischaemic stroke leading to leukoencephalopathy and Parkinson’s-like features that lasted beyond 10 weeks following an unknown snakebite has been reported from India. The patient’s Parkinson’s-like features responded well to levodopa and carbidopa combined treatment [60].

A 15-year-old male from India developed hypoxic encephalopathy following an Indian krait bite, and had paraplegia and persistent cortical blindness four years after the bite [61].

### 4.3. Blindness

Blindness and visual impairment are rarely reported following snakebites and are most commonly associated with secondary effects of envenoming. Cortical blindness has been reported in a patient with a Russell’s viper bite (*Daboia russelii*), due to an ischaemic stroke [62]. Cortical blindness has also been reported following cerebral hypoxia in a patient who had a cardiac arrest following cobra (*Naja* sp.) envenoming [63] and in a patient who developed respiratory arrest following a krait (*Bungarus* sp.) bite [61]. There are two reports of optic atrophy due to central retinal artery occlusion following suspected viper bites in India [64]. Both the patients had delayed presentations. Optic neuritis secondary to haemorrhages following a bite by *Vipera lebetina* (now *Macrovipera lebetina*) has been reported from Israel [65]. Direct venom injury from spitting cobras may also result in blindness (see above).

### 4.4. Neurological Effects following Intracranial Haemorrhage

Intracranial haemorrhage can occur in envenomings by snakes that cause venom induced consumption coagulopathy, including many vipers and Australasian elapids. In the majority of cases, intracranial haemorrhage in combination with severe coagulopathy is fatal, but some patients may survive with permanent neurological sequelae. Eight patients with *Bothrops* spp. envenoming from Ecuador were reported to have cerebrovascular events, and seven had intracranial haemorrhages [66]. Three patients survived and all had permanent neurological effects. Six patients in Australia developed intracranial haemorrhages from venom induced consumption coagulopathy following elapid envenomings, mainly brown snakes (*Pseudonaja* spp.). The only survivor was a 72-year old female who had a permanent left arm and leg hemiplegia with inattention [67]. Persistent central monoparesis of the left leg following intracerebral haemorrhage in a suspected bite by a carpet viper (*Echis* sp.) has also been reported [68].

A retrobulbar haematoma that caused raised intraocular pressure resulting in bilateral corneal opacifications has been reported in a 10-year-old child who had coagulopathy following a suspected viper (Genus: *Echis*) envenoming in Nigeria [69]. A 15-year-old Nigerian boy bitten by a carpet viper developed retrobulbar haemorrhage resulting in bilateral optic atrophy [70]. 

### 4.5. Reduced Parasympathetic Activity

Decreased parasympathetic activity following Malayan krait (*Bungarus candidus*) envenoming has been reported previously in three patients not treated with antivenom [71]. These patients developed hypertension, mydriasis and tachycardia acutely in conjunction with severe neuromuscular paralysis. While the hypertension resolved, mydriasis and tachycardia persisted for up to two years. The possible mechanisms of the autonomic effects in Malayan krait bite are poorly understood.

### 4.6. Anosmia and Changes in Taste Sensation

Changes in smell (including loss of smell—anosmia) as well as taste have been reported following Australian elapid bites, mainly for black snakes (*Pseudechis*) [72,73]. In most cases, patients report a horrible taste sensation, or change in taste/smell that persists for months to years. Persistent anosmia due to olfactory bulb atrophy has been reported in another confirmed case of a Mulga snake (*Pseudechis australis*) bite [74].

## 5. Endocrine Effects—Hypopituitarism

Clinically detectable endocrine effects are rarely reported during the acute stage of snake envenoming. Acute hypopituitarism in Burmese Russell’s viper (*Daboia siamensis*) [75], Russell’s viper in Sri Lanka (*D. russelii*) [76] and Addisonian crisis in Russell’s viper in India (*D. russelii*) [77] have been reported. The accepted pathophysiology of hypopituitarism in these cases is a haemorrhagic infarction in the pituitary resulting from venom induced consumption coagulopathy [8]. This results from the combination of the consumption coagulopathy and vascular injury from haemorrhagic toxins. Acute hypopituitarism manifests as hypotension and hypoglycaemia [75], and appears to persist, based on 11 of 12 survivors of *D. siamensis* viper envenoming [78]. The involvement of the anterior pituitary is commoner than the posterior pituitary [8].

In some snakebite survivors who had no clinically detectable hypopituitarism during the acute stage, chronic/delayed hypopituitarism may clinically manifest later as deficiency of cortisol, growth hormone, thyroxine and testosterone (in males) [8,75,79]. Two studies have summarised 36 previous cases on hypopituitarism in snake envenoming [8,80]. Almost all reported cases of chronic/delayed hypopituitarism are due to envenoming by *D. siamensis* and *D. russelii*, the majority from India. The clinical presentation includes fatigue, loss of libido, secondary amenorrhea, infertility, weight loss, hypoglycaemia and features of hypothyroidism such as facial puffiness, dry skin and cold intolerance [8,80,81]. In a large proportion of patients, necrosis of the pituitary is seen as an empty sella on magnetic resonance imaging [79,80,82] and may rarely present as psychosis [83]. 

The time to diagnosis of hypopituitarism varies from 2 weeks to 10 years [84,85]. In most studies, hypopituitarism diagnosed in a patient was linked to a snakebite that occurred several years prior, of which no reliable data existed of the acute episode. In a more recent cohort study from India, 60 patients had baseline hormonal profiles immediately after the snakebite and were prospectively followed for six months [82]. Of them, six patients developed asymptomatic anterior hypopituitarism during the acute period following the bite. All of them had deficiencies in growth hormone, gonadotrophin, thyroid hormones and secondary adrenal insufficiency. However, none of the other 54 patients developed hypopituitarism in the acute period or at six months. This clearly indicates that the initial insult to the anterior pituitary occurs during the acute stage of envenoming, while the patients are asymptomatic. However, the damage is irreversible, and hypopituitarism persists with subsequent progression to clinically detectable hypopituitarism. 

Most of the above studies used less reliable methods of case-authentication, or assumed the identity of the biting species, which might have limited the generalisation of these findings.

## 6. Psychological Effects

Psychological sequalae are important, but greatly under reported effects of snake envenoming. These effects are unlikely to be direct venom effects, but rather the effects triggered by the traumatic experience of a snakebite and the severe socioeconomic consequences associated with snakebite. In a cross-sectional study done in Nigeria, depression was prevalent in 25% of 187 snakebite patients who were receiving treatment in the hospital [86]. The depression was associated with more severe complications in snakebite, being worried about family welfare, time and financial loss and previous experiences with snakebite.

In a study from Sri Lanka that included both quantitative and qualitative arms, 88 patients 12–48 months after the snakebite had significantly higher rates of depressive symptoms, post-traumatic stress disorder and somatisation symptoms, compared to the matched controls who had no history of snakebite [11]. In this study, 54% of patients had depressive symptoms as opposed to 13% in the controls. The qualitative study found various unexplainable elements of somatisation such as blindness, tooth decay, body aches, headaches, tiredness and weakness in snakebite survivors. 

Following this study, a randomised controlled trial of a brief psychological intervention was undertaken in Sri Lanka. The intervention included psychological first aid, psychoeducation and cognitive behavioural therapy, which was associated with a reduction in psychiatric symptoms and disability in snakebite victims, compared to controls [87]. However, the intervention was not effective in preventing depression or post-traumatic stress disorder.

## 7. Knowledge Gaps

Snakebite is underreported because most affected people are poor, rural, in politically disadvantaged communities, even within their own countries [1,88]. Modern medicine is still not available for many snakebite victims in the rural tropics. For these reasons, the epidemiology, clinical effects, consequences and socioeconomic impact of snakebite are still poorly understood [89]. Many of those treated in hospitals for the snakebite are likely to not attend follow-up. Even in countries such as Sri Lanka, in which the vast majority of patients seek western medicine as the first choice for snakebite [90], patients seek treatment from the local indigenous doctor for persisting symptoms, without being directed to rehabilitation programs for their musculoskeletal disabilities [9]. Even in developed settings, long-term issues related to envenoming in snakebite victims are poorly addressed or reported [28]. The few existing studies of the long-term effects of snake envenoming are based on selected patient groups for follow-up, hence do not provide the true picture of the burden. Some studies have described long-term effects of snake envenoming by relating a disability to a previous snakebite, based on the patients’ interpretations, which might be biased. The community-based studies on the long-term effects of snake envenoming are useful in understanding the burden of long-term effects of snake envenoming in general. However, they are unable to answer the clinical questions due to the inherent deficiencies associated with recall bias, so there is less-reliable information on the acute stage of envenoming. 

The range of clinical effects and their severity in snake envenoming are unique for individual snake species. Therefore, accurate species identification is essential in clinical and epidemiological studies. This can be done by either identification of the snake specimen by a herpetologist or specific venom detection enzyme-linked immunosorbent assay (ELISA) [91]. Most of the studies that describe the long-term effects of snakebite did not have accurate species authentication, which has limited the interpretation of the results. Cohort studies that document the acute stage of envenoming must have accurate case-authentication and active follow-up of patients on regular intervals to provide a detailed picture of the epidemiology and the clinical consequences of the long-term effects of snake envenoming. In regions where the facilities are available, implementation of institutional databases that record the details of the acute envenoming as well as the details of the follow-up visits of the discharged patients would be useful. 

From the available studies, it appears that the socioeconomic burden resulting from the physical and psychological consequences of delayed and long-term effects of snake envenoming is enormous. Management guidelines of snakebites are largely focused on the acute management of snakebite. At present, the attention paid to following up snakebite patients for psychological effects is non-existent. In reality, even for minor effects of local necrosis, proper follow-up for physical therapy and exercises does not happen due to patient and health-system factors, and hence end up as physical disabilities due to contractures, which are preventable [10]. In the future, management protocols for snakebite must address the issues related to long-term effects of snake envenoming. 

## 8. Methods

**Figure 2:**
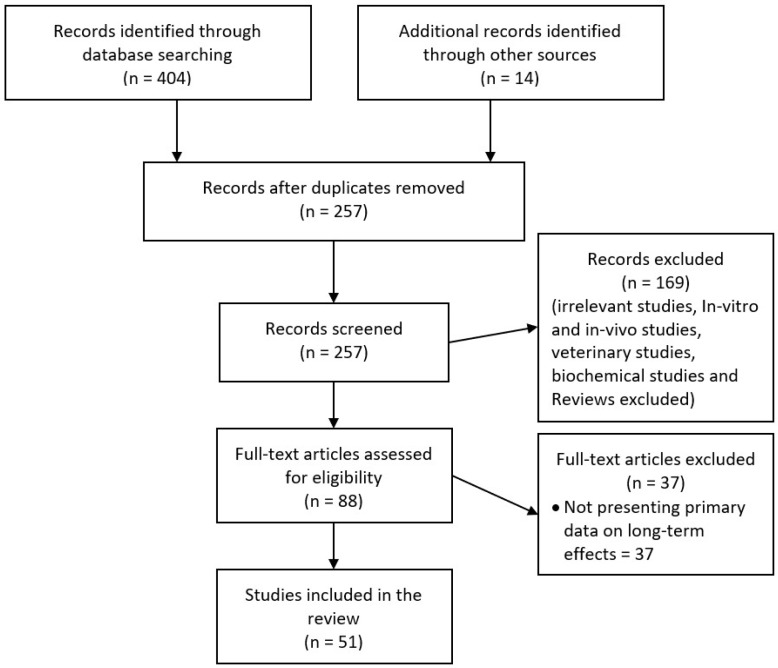
Selection of studies for the review.

We carried out a search in MEDLINE from 1946 and EMBASE from 1947 to 14 October 2018 and included clinical studies in English on snakebite that describe long-term or chronic effects. We used the search terms “snake envenoming”, “snake envenomation”, “snake bite”, and “ophitoxaemia” in combination with the terms “chronic”, “long term”, “delayed”, “prolonged”, “disability”, “persistent”, “permanent”, “morbidity”, and “recurrence”. We then searched the reference lists of retrieved articles for additional publications relevant to the topic. This search yielded 404 abstracts. After removing duplicates and non-clinical studies, we identified a total of 88 abstracts for further study and the full articles of these were reviewed. From these, we excluded 37 studies that were not presenting primary data and the remaining 51 studies were included in this study (Figure 2). For this review, we classified conditions that last or appear more than six weeks following envenoming as long term or delayed effects of envenoming. We identified different themes/effects of long-term consequences of the snake envenoming based on the content of the included articles.
